# Neural Fourier Energy Disaggregation

**DOI:** 10.3390/s22020473

**Published:** 2022-01-09

**Authors:** Christoforos Nalmpantis, Nikolaos Virtsionis Gkalinikis, Dimitris Vrakas

**Affiliations:** School of Informatics, Aristotle University of Thessaloniki, 54124 Thesssaloniki, Greece; nvirtsion@csd.auth.gr (N.V.G.); dvrakas@csd.auth.gr (D.V.)

**Keywords:** non-intrusive load monitoring, energy disaggregation, nilm, deep learning, fourier, neural fourier

## Abstract

Deploying energy disaggregation models in the real-world is a challenging task. These models are usually deep neural networks and can be costly when running on a server or prohibitive when the target device has limited resources. Deep learning models are usually computationally expensive and they have large storage requirements. Reducing the computational cost and the size of a neural network, without trading off any performance is not a trivial task. This paper suggests a novel neural architecture that has less learning parameters, smaller size and fast inference time without trading off performance. The proposed architecture performs on par with two popular strong baseline models. The key characteristic is the Fourier transformation which has no learning parameters and it can be computed efficiently.

## 1. Introduction

Non-intrusive load monitoring (NILM) is a growing research subject and is believed to have a large impact on energy conservation. The benefits include energy awareness, identification of faulty appliances, improved building operational efficiency, more accurate energy consumption forecasting and others [1]. The goal of NILM is to disaggregate the energy that is consumed in a household and is also known as power or energy disaggregation. It is a blind-source separation problem and is classified as NP-hard [2].

Modern NILM systems are based on deep learning, where one neural network is given the total energy consumption of a house and the target is the energy of a single appliance. Recognizing many appliances with one model has attracted the interest of many researchers as well. Multi-label approaches usually identify on-off states of a predefined number of appliances [3,4]. This research focuses on the single regression approach, aiming to develop a computationally efficient energy disaggregator.

In addition to the computational difficulty of the disaggregation problem, there are several parameters that affect an experimental environment. These include differences among datasets, the sample frequency of the energy data, the time-frame that a prediction occurs, the number of active devices etc. The complexity of the environmental setup makes the reproducibility of NILM experiments a hard task. In order to overcome the comparability issue, Symeonidis et al. [5] propose a benchmark framework that describes different scenarios of testing NILM algorithms. Batra et al. [6] try to tackle reproducibility issues by providing the implementation of nine different disaggregation algorithms along with state-of-the art experimental results. Despite the aforementioned efforts a widely accepted standardization of comparing NILM systems is still pending [7].

The contribution of this research is threefold. The first contribution is the development of a novel architecture, that incorporates the Fourier transform and is called neural Fourier energy disaggregator (NFED). It is inspired by FNet [8], where Fourier transform is used as a faster alternative to attention mechanism. The second contribution is an ablation study comparing two versions of the proposed neural network. One version is the suggested that uses Fourier transform and the other one replaces the Fourier transform with the attention mechanism. The third contribution is a comprehensive comparative analysis that aims to find the best model per appliance through an extensive tuning methodology that takes into account both experimental and architectural hyper-parameters. The models that are compared are NFED, window-GRU (WGRU) [9], sequence-to-point (S2P) [10] and self-attentive energy disaggregator (SAED) [11]. For a fair comparison the best environmental setup is found for each of the four models that are compared. Then, utilizing the benchmark framework of Symeonidis et al. [5], it is demonstrated that the proposed model achieves close to state-of-the-art results, whereas it remains computational efficient, it has less learning parameters and requires relatively small storage space.

## 2. Related Work

Deep learning has shown unprecedented performance in several domains spanning computer vision, natural language processing (NLP), sound recognition and time series analysis. Their application in NILM was firstly introduced by Kelly and Knottenbelt [12]. The authors proposed three architectures including a recurrent neural network based on long short-term memory (LSTM), a denoising autoencoder and a convolutional neural network which predicts the start and end time along with the power demand of each appliance. These architectures were evaluated on the UK-DALE dataset [13] outperforming previous FHMM approaches.

Nowadays, NILM researchers focus on deep neural architectures. Despite the large variety of different neural components there is no evidence that a specific architecture fits better the problem of power disaggregation. In the literature the most common architectures are variants of recurrent neural networks [9,14,15,16] or based on convolutional layers [10,17]. Denoising autoencoders are also very popular and most of the times their first layers are convolutional [18,19]. Models based on the attention mechanism demonstrate promising results in terms of generalization to unseen data. The attention mechanism is incorporated using the self-attention method [11,20,21] or the transformer architecture [22]. Recently, generative models have been proposed for the problem of NILM by using GANs [23] or variational approaches [24,25,26]. For the reader’s reference, Huber et al. [27] present an extensive review of several deep learning solutions for NILM.

A different approach of the problem, with the aim to reduce computational resources, is the technique of transfer learning [28,29]. Kukunuri et al. [30] suggest to apply compression methods to reduce the size of deep neural networks so that they fit on edge devices. The method that they propose is a multi-task based on parameter sharing. Deploying NILM models into embedded devices that require real-time inference, is an emerging research direction [31,32]. Athanasiadis et al. [33] develop a multi-class NILM system that can detect any number of appliances in real-time. The system can be embedded into simple microprocessors. The key component of the proposed method is the processing of measured turn-on active power transient responses sampled at 100 Hz.

The aim of this work is to build an efficient neural architecture that has high inference speed and requires low storage. A novel neural architecture is developed, named neural Fourier energy disaggregator (NFED). NFED occupies less capacity because of the reduced learning parameters, while it performs on par with other state-of-the-art NILM systems. Furthermore, a detailed hyper-parameter tuning is conducted based on the factors that affect the performance of NILM models. One of the most important factors is the size of the window which depends not only on the target appliance but also on the architecture of the model. The final experiments take the window size into account and all the models are adjusted finding their best performing window per appliance. The evaluation is based on the benchmark framework that is proposed by Symeonidis et al. [5], showcasing that the proposed architecture performs on par with other strong baselines, whereas it has less learning parameters, faster inference and training time and reduced size.

## 3. Materials and Methods

### 3.1. Datasets

The experiments of this work are based on three public datasets: UK-DALE [13], REDD [34] and REFIT [35]. UK-DALE and REFIT contain data from UK and REDD from the USA. REFIT includes 20 houses and a wider range of devices. Five household devices are used to evaluate the disaggregation models: dish washer (DW), fridge (FZ), kettle (KT), microwave (MW) and washing machine (WM).

### 3.2. Preprocessing

The preprocessing step is very simple because neural networks accept raw data as input. It is very important to align the input and the target in terms of date and time. Furthermore, the datasets might have some missing values which are replaced by zeros. The main step of preprocessing is to standardize the data using the following formula:

(1) $$Z=\frac{x-\mu }{\sigma }$$

where *Z* is the standard score, *x* the observations, μ the mean of the sample and σ the standard deviation. The standardization of the target appliance is adjusted accordingly by calculating the statistics of its energy consumption. Had the same statistics been used, the standardized target might have taken tiny values.

### 3.3. Methodology

The experiments of this work are conducted in four steps, from the development of the proposed model to its comparison against existing ones. The four experimental levels are described in Table 1. The first step after developing the architecture of NFED is to tune its hyper-parameters and find the best depth and number of neurons. The experiments use 5 cross validation evaluating variations of NFED on UK-DALE house 1. In the case that two different versions of the model are equal, the computationally lighter is preferred.

**Figure 1:**
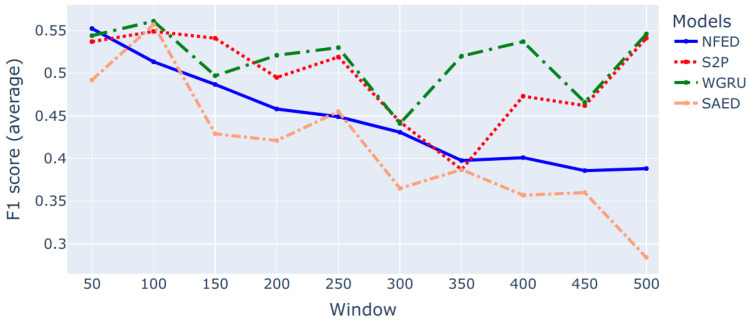
An example of how the performance of various models is affected by the input length. The target appliance is a washing machine and the evaluation metric is F1 score.

The second step is to adjust the parameters of the environment for each model separately. The most important parameter is the size of the input sequence. The best window length for each model is decided via a series of experiments that use the 5 cross validation technique for each target appliance. The final configuration of window length per appliance for each model is presented in Table 2. A representative example of this type of experiments is depicted in Figure 1. The $F_{1}$ score is the average score of the 5 cross validation iterations. As shown in the figure, the lightweight models SAED and NFED perform better with small windows for the case of a washing machine. The larger models S2P and WGRU show a decline in performance while the window size is increased and then after window length 350 the performance increases again. The maximum window that is tried is 500 samples, which corresponds to 50 min.

The last two steps of our methodology concern the application of the benchmark framework for two variations of the proposed neural network and for a comparative analysis of NFED against three other models. The ablation study aims to clarify the benefits of using the Fourier transformation against the attention mechanism as an alternative method. The main advantage of Fourier transform is lower computational complexity, faster inference speed and smaller size of a trained model. Regarding the evaluation and comparison of the proposed model against existing ones, the process aligns with the benchmark framework that is proposed by Symeonidis et al. [5] and includes four basic scenarios. In the first case the models are trained and tested on the same house at different time periods. The test data are chronologically after the training data. Therefore, little or no distribution shift is expected. Models with low performance in these experiments are considered weak because this is the easiest evaluation case. In the second scenario a distribution shift of the data is expected, because test data belong to different houses which are not seen during training. The different energy consumption patterns can be attributed to the habits of the residents and the variety of appliances. The third and fourth scenarios consider the learning capabilities of the models across many buildings and testing on the same and different dataset. The four categories of the benchmark are summarized as follows: single building NILM, single building learning and generalization on the same dataset, multi building learning and generalization on the same dataset and generalization on a different dataset. Table 3 presents the details of the datasets and the corresponding houses that are selected for each category of the benchmark framework.

### 3.4. Evaluation Metrics

The most common metrics when evaluating the performance of a NILM system are $F_{1}$ score and mean absolute error (MAE). $F_{1}$ score corresponds to the detection of whether a specific appliance is consuming energy. It is computed using Equation (2) which is the harmonic mean of Precision and Recall. Precision and Recall are described in Equations (3) and (4), respectively. MAE measures how much the predicted power consumption diverges from the real one. It is measured in Watts and its equation is described by (5) where *T* is the length of the predicted sequence, $y_{t}’$ the estimated electrical power consumption and $y_{t}$ the true value of active power consumption at moment *t*.

(2) $$F_{1}=2\frac{Precision\times Recall}{Precision+Recall}$$

(3) $$Precision=\frac{TP}{TP+FP}$$

(4) $$Recall=\frac{TP}{TP+FN}$$

(5) $$MAE=\frac{1}{T}\sum \;|y_{t}^{\prime }-y_{t}|$$

The benchmark framework that is utilized in this work, includes testing on unseen data as well. In order to quantify the generalization capabilities of the models the metric of generalization loss (G-loss) is used [36]. The G-loss is calculated by Equations (6) or (7), depending on whether the basic metric is $F_{1}$ or MAE. The index *u* stands for unseen and *s* for seen data. The higher the G-loss the worse the generalization. The average generalization performance can be calculated using the mean generalization loss (MGL) according to Equation (8). Furthermore, the average $F_{1}$ score and the average loss are also taken into consideration using Equations (9) and (10).

(6) $$G-loss=100(1-\frac{F1_{u}}{F1_{s}})$$

(7) $$G-loss=100(\frac{MAE_{u}}{MAE_{s}}-1)$$

(8) $$MGL=\frac{1}{N}\sum_{i}^{N}\;G-loss_{i}$$

(9) $$AUH=\frac{1}{N}\sum_{i}^{N}\;F1_{ui}$$

(10) $$EUH=\frac{1}{N}\sum_{i}^{N}\;MAE_{ui}$$

## 4. Architecture of Neural Networks

In the literature, there are several neural architectures that are proposed for the problem of NILM [27]. Unfortunately, very few research papers are supported with source code, many are missing critical details and some are tested on private datasets. To overcome the aforementioned reproducibility issues, the baseline models are selected based on how easy it is to replicate past experimental results, their wider acceptance by other NILM researchers and the existence of implementations in open source projects such as NILMTK [6,37]. The baseline models are: a convolutional neural network named “sequence-to-point” (S2P) [10], a recurrent neural network named “online GRU” or “window GRU” (WGRU) [9] and a neural network based on the self-attention mechanism named “self-attentive energy dissaggragator” (SAED) Virtsionis-Gkalinikis et al. [11]. The first two models have been used either as baselines or as a basis to develop new architectures. They are also part of the NILMTK toolkit and consist two very strong baselines. SAED is a relatively new architecture but has shown good results and is computationally light. It has strong generalization capabilities and can be used as a baseline for computationally lightweight models with very few learning parameters. The details of these neural nets are presented in the next subsection.

### 4.1. Baseline Models

**Figure 2:**
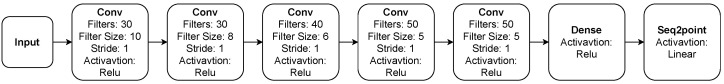
Architecture of S2P.

Sequence-to-point (S2P) is a convolutional neural network proposed by Zhang et al. [10]. The original architecture of the network accepts as input a sequence with size 599. It consists of five convolution layers with the non-linear activation function ReLU. The final layer is the output of a linear activation function. The details of the layers are depicted in Figure 2.

**Figure 3:**
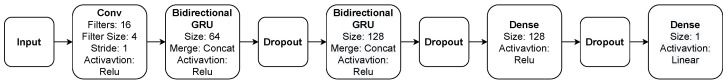
Architecture of WGRU.

Window GRU (WGRU) is introduced by Krystalakos et al. [9] and its main component is the recurrent layer GRU [38]. The first layer is a convolutional one, followed by two bidirectional GRU layers and one dense layer before the output. In order to prevent over-fitting, the dropout technique [39] is used between layers. The input is a look back sliding window. Figure 3 shows the details of the architecture.

**Figure 4:**
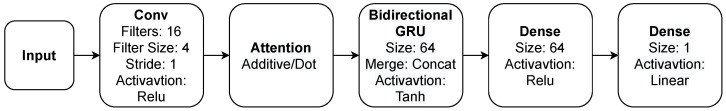
Architecture of SAED.

Self-attentive energy disaggregator (SAED) is based on the mechanism of attention and is developed by Virtsionis-Gkalinikis et al. [11]. It is a computationally efficient neural network. It is trained up to 7.5× faster than WGRU and its inference time is up to 6.5× faster. The architecture includes a convolutional layer, followed by the attention mechanism. There are two variations of the attention mechanism the additive or dot attention. Next, there is a bidirectional GRU layer and finally a dense layer. Figure 4 illustrates the overall architecture.

### 4.2. The Proposed Fourier Based Neural Architecture

The transformer architecture [40] has demonstrated state of the art results in NLP and computer vision. Its success is mainly attributed to the Attention mechanism [41]. The models that utilize the Transformer architecture are capable of understanding the context of the given input and focus on the features that are important. In terms of computational performance, the attention mechanism provides faster processing than recurrent neural networks because of parallelization. Researchers have tried to further improve the performance of attention to build faster Transformer architectures [42,43,44,45]. Recently, Fourier transform has been proposed as an alternative to attention mechanism, by replacing it within the Transformer architecture [8]. The latter architecture is called FNet and the main benefit is that Fourier transform does not have any learning parameters. For the computation of Fourier transform the fast Fourier transform (FFT) is employed. The equation that describes the discrete Fourier transform of the complex numbers $x_{0},x_{1},\ldots x_{N-1}$ is given as follows:

(11) $$X_{k}=\sum_{N-1}^{n=0}x_{n}e^{-i2\pi kn/N}$$

where k=0,1,…N−1. Computing directly the discrete Fourier transform requires $O(N^{2})$, however most implementations that use the FFT algorithm require O(NlogN).

**Figure 5:**
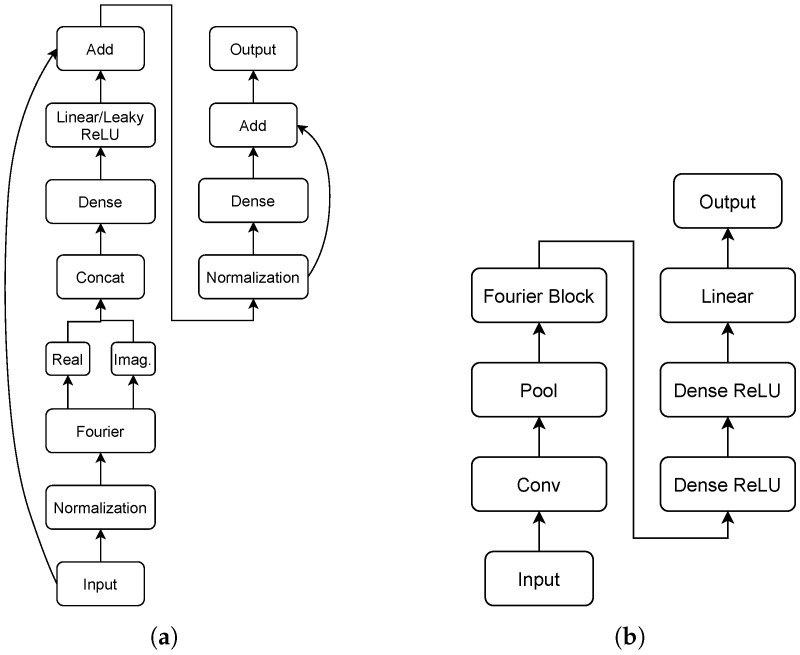
The proposed NFED neural network and the Fourier block architecture. (**a**) Fourier block; (**b**) NFED architecture.

This work proposes a novel neural architecture, named neural Fourier energy disaggregation (NFED). To the best of the authors’ knowledge, it is the first time that a Fourier based neural network is suggested for the problem of NILM. The basic component of the network is an architecture called Fourier block, which is illustrated in Figure 5a. The input of the block is a tensor, which is firstly normalized. Then the Fourier transform is applied. The real and imagine parts are concatenated and pass via a dense layer. The activation function is a linear or a leaky relu. It is noticed that for some appliances such as dish washer, leaky relu boosts the performance of the model. Next, the original input is added with the output of the dense layer via a residual connection. There is another normalization layer, followed by a linear dense layer. The input to the dense layer is added as a residual connection to its output, giving the final output of the block. The entire architecture of NFED is depicted in Figure 5b. It includes a convolutional layer, followed by a 1D power-average pooling operation. Next, there is a Fourier block and its output goes through two non linear dense layers with relu activation function. Finally, a linear layer gives the output of the network.

As it is described previously, a second version of NFED is developed based on self-attention. The attention mechanism is very popular in modern neural architectures such as transformers [40]. However its computational complexity lead to the quest of alternatives. In this paper, the proposed architecture is used as a case study that examines if Fourier transform can replace attention for the problem of NILM. Therefore, a second version of NFED is the one where Fourier transform is substituted by self-attention. More details about the comparison of the two versions of NFED are presented later in this paper in the context of an ablation study. The proposed architecture and the various versions are implemented in pytorch and code is availabe at https://github.com/ChristoferNal/Neural-Fourier-Energy-Disaggregation, accessed on 5 January 2022.

### 4.3. Properties of the Neural Disaggregators

For each pair of appliance and model there is a specific input length that improves the performance. The properties of the models are affected not only by the architectural design, but also by the input length. For a recurrent neural network a long input means longer training and inference time. For a fully connected network, a large input would affect the number of its parameters and thus performance and speed.

Table 4 presents the detais of the final models that have been designed in this research for five devices. The benefit of the proposed architecture is that for the majority of the appliances, NFED performs well with a relatively small window size. For example, for kettle and microwave the window consists of 50 values, which is the smallest window. The large models, WGRU and S2P, in general perform better with larger windows. This is usually more than double of the window of NFED or SAED. All the models, apart from WGRU, are affected in terms of the learning parameters, which are increasing when the window increases. WGRU maintains the same number of learning parameters, regardless of the window. On the other hand WGRU is heavily affected in terms of training and inference speed, because it processes the input data sequentially. SAED overcomes this problem because it has only one recurrent layer and the inference speed is not affected heavily. The fastest models in terms of average training speed are SAED and S2P. NFED is very close to the other two and sometimes faster. Initially, this looks counter intuitive because S2P has much more learning parameters than any other model. One of the underlying reasons is that the majority of its layers are convolutional ones, which are computed very fast in modern GPUs and the depth is slightly smaller than NFED.

Two significant properties of neural networks, especially when deploying them on the edge, are the size of the model and the inference time on a CPU. These two properties are important because of the limited resources of edge devices. Table 4 includes the size and the inference speed of the models when they run on a CPU. The smallest model is SAED, which is less than a half megabyte. WGRU requires 2.794 MB regardless of the target appliance that is recognized. NFED requires 1.8MB for the cases with very small window such as kettle and microwave. It can take up to 17.336MB which is the case for dishwasher. Finally, S2P is the largest model and its size ranges from approximately 20 MB to 102 MB. The two smallest models are suitable for deployment on devices with limited storage but we have to take into account their disadvantages. SAED trades off a lot of performance and WGRU can be very slow due to the recurrent units. If the speed is not an issue then WGRU is a good solution as its performance is equivalent with its competitors. If there are strict requirements on storage, efficiency and performance, then NFED is the most suitable model. It can be up to 34× smaller in size than S2P without trading off performance and has low latency when it is run on a CPU.

## 5. Experimental Results and Discussion

In this section, three types of experiments are described. An ablation study is conducted to explore two different versions of the proposed architecture and scrutinize the benefits of Fourier transformation against self-attention. Next, there is a meticulous comparative analysis of the performance of four neural networks on the problem of energy disaggregation. Finally, there is a discussion on the experimental results that explains which neural network should be selected for specific case studies, as a model can be a good fit depending on the requirements.

### 5.1. Ablation Study

One of the goals of this research is to highlight the differences in performance and computational requirements, between the Fourier transform and self-attention mechanism, when interchanged in the proposed neural architecture. The comparison is executed in the first two categories of the followed benchmark methodology using $F_{1}$ score. The two variations of the network are also evaluated in terms of storage size, train and inference speed.

As shown in Table 5, the attention variant model (ATT) occupies larger size in memory than the proposed model (FFT) as expected. Furthermore, smaller train and inference speeds are achieved. Despite the attention model being slower, the network contains only one layer and the differences in speeds are not significant, especially on the GPU.

**Figure 6:**
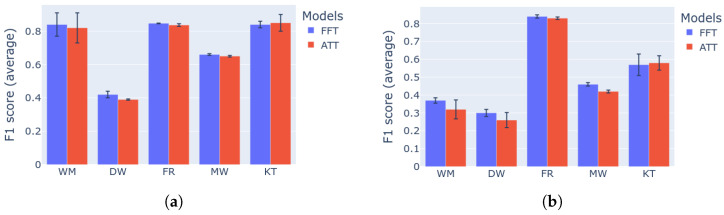
Benchmark results for the ablation study: (**a**) category 1: single building NILM; (**b**) category 2: single building learning and generalization on the same dataset.

In terms of the performance, the comparison is summarized in Figure 6. In overall, the proposed NFED model performs better and with smaller standard deviation than the attention variation, for the majority of the appliances. Thus, in the context of the novel neural network structure, the FFT seems to outperform the self-attention mechanism. Interestingly, the difference in performance is more notable in the category 2 of experiments, highlighting good generalization capabilities of FFT.

### 5.2. Comparative Performance Analysis

**Figure 7:**
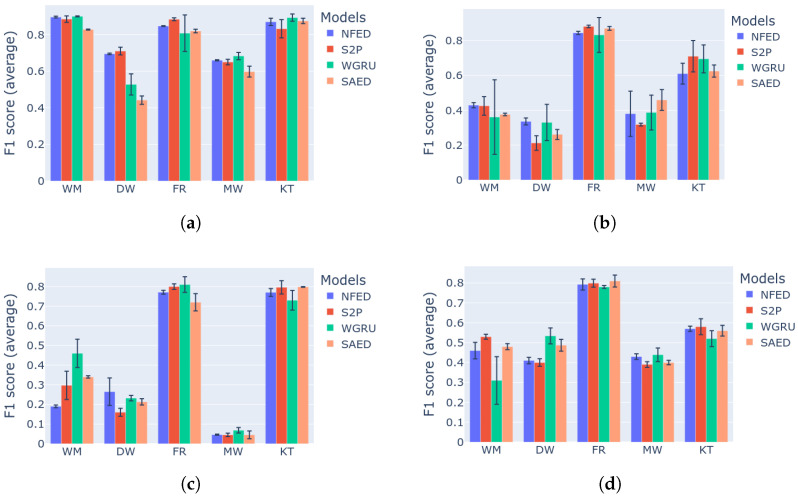
Benchmark results for the models NFED, S2P, WGRU and SAED. (**a**) Category 1: Single building NILM: (**b**) category 2: single building learning and generalization on the same dataset; (**c**) category 3: multi building learning and generalization on the same dataset; (**d**) category 4: generalization on a different dataset.

**Figure 8:**
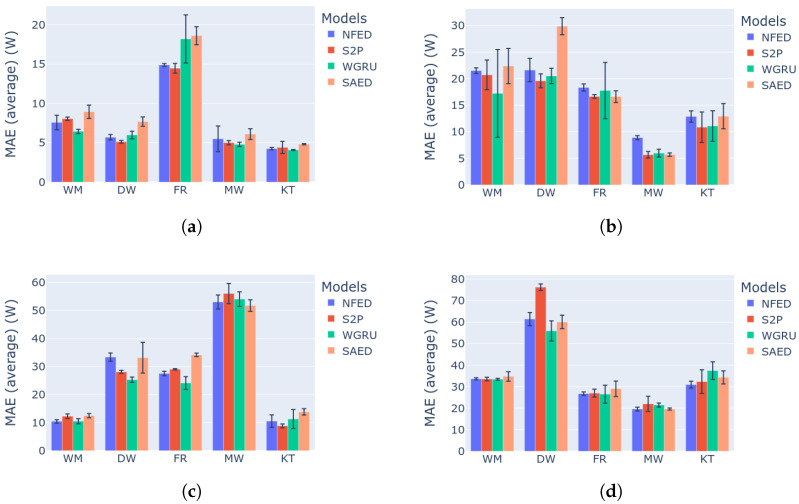
Benchmark categories 1–2 results for the models NFED, S2P, WGRU and SAED: (**a**) category 1: MAE results; (**b**) category 2: MAE results. Benchmark categories 3–4 results for the models NFED, S2P, WGRU and SAED: (**c**) category 3: MAE results; (**d**) category 4: MAE results.

NFED is evaluated and compared against two strong baseline models, S2P and WGRU. These two models achieve high $F_{1}$ score and low MAE. The disadvantages are that S2P has a large number of parameters, which means that a trained model has a relatively large size. WGRU does not have many parameters but it is slow because it mainly consists of recurrent units that do serial computations instead of parallel ones. There is a third baseline model, named SAED, which is a weaker disaggregator but very lightweight and achieves good generalization performance due to the attention mechanism. Utilizing the benchmark framework that is described in previous sections, the four models are evaluated and compared for the following appliances: dishwasher, washing machine, fridge, kettle and microwave. The evaluation metrics are $F_{1}$ score and MAE. Figure 7 presents the results regarding the $F_{1}$ score and is analyzed in this section. The results for MAE are similar and for reference are presented in Figure 8.

Starting with the first category of experiments, which evaluates the models on unseen future data of the same house that was used for training, the proposed model achieves the best or second best $F_{1}$ score for all the devices. From Figure 7a it is evident that, NFED not only is consistent in performance, but it also shows the smallest standard deviation across many repetitions of the same experiment. Similar results are demonstrated for the second category of experiments as shown in Figure 7b, where the test data come from a different house. For these two categories of experiments, NFED is very competitive disaggergating the appliances dish washer and washing machine. S2P and WGRU follow, with S2P having smaller standard deviations, but has the worst performance for the case of dish washer on category 2. Regarding the fridge all four models perform well, with S2P and NFED taking the first and second place with small difference. As far as the microwave is concerned WGRU and NFED are the best models on category 1 and loose the first place by SAED on category 2. This can be attributed to the strong generalization capabilities of SAED. Regarding kettle on the first category all the models achieve more than 80% $F_{1}$ score. There is performance degradance on category 2 with S2P and WGRU performing the best, followed by NFED and SAED.

The last two categories of the benchmark are the hardest tasks. In category 3 a model tries to learn from many buildings, which is not a trivial task, as there might be more patterns to learn. The two training houses might have different number of appliances with different energy consumption footprint. Then testing occurs on an unseen house with different energy consumption footprint. Therefore, the model is prone to learn the common characteristics of the two training houses and testing is based on these learnt representations. The intuition is that the common patterns of three different houses are equal or less than the common patterns of two houses. In the same fashion, category 4 is even more difficult as the test data come from a different electricity grid. Despite category 4 being in general more difficult, the final result depends heavily on the actual complexity of the testing house, e.g., how many appliances it has. Overall, for both multi-building training categories, the proposed model achieves better or equal performance with the other models. SAED presents strong generalization because it demonstrates low performance reduction from the single building cases. More details on the generalization performance of the models can be found in Appendix A. The two strong baselines, S2P and WGRU are competitive but none of them is consistently a top performer. Because of the complexity of these particular tests, it is not easy to select the best model for a specific appliance. From Figure 7c,d the safe conclusion that can be made is that NFED performs on par with the baselines. Overall, the two best performing models are NFED and S2P.

### 5.3. Selecting the Right Model for a NILM System

Comparing NILM models is difficult because of the complexity of the problem. In the real world, most of the times testing data are out-of-distribution. This is a fundamental open problem in machine learning. The benchmark framework that is used in this research simulates the aforementioned problem and the results show that no model is capable of performing in the same way in out-of-distribution data. Furthermore, in the real world it is equally important to take into account the properties of the model such as its size and how fast it can run on different computing resources.

**Figure 9:**
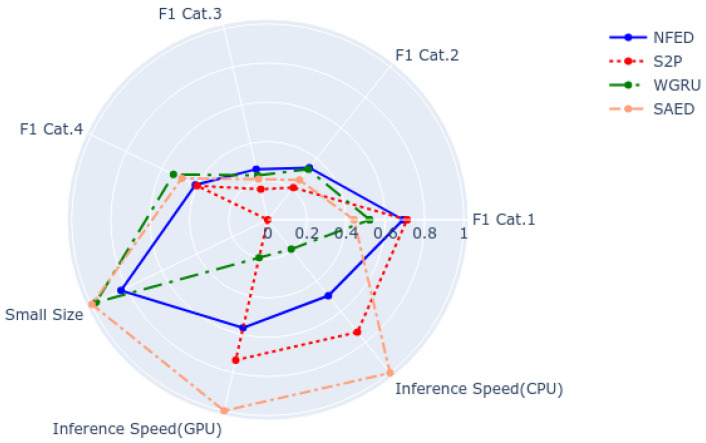
Diagram that summarizes the capabilities of the models to disaggregate a dish washer.

A fair way to compare different NILM models is to consider all the experimental results of the benchmark and the properties of the models. Figure 9 is a representative example for dish washer. It includes the $F_{1}$ score for the four categories of the benchmark and the following properties of the models: their size, the inference speed on a GPU and the inference speed on a CPU. All the attributes have their best values on the outer space of the disk. The closer to the centre the worse the result. As it is observed, NFED achieves top performance for all four categories and thus it is a good candidate to be deployed in the real world. Looking at the rest of the properties, NFED is the third fastest model. S2P would be a good candidate it speed is critical without sacrificing much performance. On the other hand, S2P has the largest size by far, which would make it prohibitive if there are strict constraints regarding storage space. All five appliances that are used in this paper are analyzed in the same way in Appendix B. To conclude, each model has each advantages and disadvantages and can be a good fit depending on the significance of the parameters and the requirements of the overall system.

## 6. Conclusions

Designing a non-intrusive load monitoring system can be very complex. The system requirements can vary depending on the target environment where the model will be deployed. Running energy disaggregation models on the cloud can be more flexible because of the plethora of resources. On the other hand a cloud solution can be very costly when the systems scales up. The alternative is to run such models on an embedded device, where the resources are limited. This manuscript proposes a novel neural network, named NFED, which is suitable for both solutions. NFED requires relatively small size, it has fast inference speed and achieves similar or better results in terms of performance. The key of NFED’s efficiency is the incorporation of Fourier transform, which can be computed fast and does not have any learning parameters.

For future work, Fourier transformation is advised to be used in more architectures, especially if the models are targeted for edge devices. In addition to Fourier, wavelets are suggested to be explored within NFED or another neural architecture. Wavelet transformation has the advantage to contain more information about time, whereas Fourier transformation provides information only in the frequency domain. Researchers should not only conduct experiments on specific datasets, but also evaluate new models using a benchmark framework. NILM solutions should be compared considering specific case studies and taking into account all the requirements including the performance and all the properties of a model.

## Figures and Tables

**Table 1:** Summary of experiments.

Experiment	Environment Setup	Goal
Hyper parameter tuning of the proposed architecture (NFED).	5 CV on house 1 from UK-DALE.	To select the best hyper parameters of NFED considering the number of the neurons and the depth of the network.
Tuning of input length per appliance for each model.	5 CV on house 1 from UK-DALE.	To find which window length achieves the best performance for each model, given a target appliance.
Ablation study comparing Fourier transform and self-attention mechanism.	Follow the four categories of experiments of Symeonidis et al. [5].	To compare the effectiveness of Fourier transform within the proposed neural architecture against the mechanism of attention.
Model evaluation using a benchmark framework.	Follow the four categories of experiments of Symeonidis et al. [5].	To evaluate and compare the performance of the proposed model against the baselines.

**Table 2:** A map of the models under evaluation with the respective best window length for each appliance.

	Microwave	Kettle	Fridge	Washing Machine	Dish Washer
NFED	50	50	350	150	450
S2P	100	300	400	400	500
WGRU	100	150	450	150	350
SAED	100	50	250	50	200

**Table 3:** Buildings used for train and test. In categories 1–3, UK-DALE was used for both training and testing. In Category 4, UK-DALE was used only for training. For testing DW and KT REFIT was used, whereas REDD was used for testing FZ, MW and WM.

Device	Category 1		Category 2		Category 3		Category 4	
	Train	Test	Train	Test	Train	Test	Train	Test
DW	1	1	1	2	1, 2	5	1, 2	2
FZ	1	1	1	2	1, 2, 4	5	1, 2, 4	3
KT	1	1	1	5	1, 2, 4	5	1, 2, 4	2
MW	1	1	1	2	1, 2	5	1, 2	1
WM	1	1	1	4	1, 5	2	1, 5	3

**Table 4:** Properties of the tested models for each appliance. Number of parameters, size of the model per device, training speed (GPU), inference speed (GPU and CPU).

Device	Model	Window	Params	Size (MB)	Train (it/s)	Inference GPU (it/s)	Inference CPU (it/s)
DW	NFED	450	4.3 M	17.336	32.14	92.36	51.44
	S2P	500	25.6 M	102.597	34.93	120.03	76.10
	WGRU	350	698 K	2.794	10.17	32.38	19.90
	SAED	200	**119 K**	**0.480**	**36.72**	**163.29**	**103.76**
WM	NFED	150	1.4 M	5.444	42.63	119.25	115.13
	S2P	400	20.5 M	82.117	24.34	87.96	87.21
	WGRU	150	698 K	2.794	14.60	45.87	45.64
	SAED	50	**44.9 K**	**0.180**	**66.04**	**270.48**	**269.63**
FZ	NFED	350	3.3 M	13.212	23.69	64.39	65.19
	S2P	400	20.5 M	82.117	**40.89**	**139.25**	**87.27**
	WGRU	450	698 K	2.794	8.02	25.01	15.39
	SAED	250	**164 K**	**0.660**	37.79	**141.19**	85.19
KT	NFED	50	449 K	1.8	**76.12**	**181.70**	193.77
	S2P	300	15.4 M	61.637	31.86	114.12	113.30
	WGRU	150	698 K	2.794	14.47	47.31	47.24
	SAED	50	**44.9 K**	**0.180**	64.97	291.71	**286.95**
MW	NFED	50	449 K	1.8	75.71	174.99	**172.71**
	S2P	100	5.21 M	20.677	**78.31**	**206.68**	162.30
	WGRU	100	698 K	2.794	21.71	66.58	66.42
	SAED	100	**59.9 K**	**0.240**	56.05	179.75	150.87

**Table 5:** Properties of the ablation study models for each appliance. Number of parameters, size of the model per device, training speed (GPU), inference speed (GPU).

Device	Model	Params	Size (MB)	Train GPU (it/s)	Inference GPU (it/s)
DW	FFT	4.3 M	17.336	32.14	92.36
	ATT	4.7 M	18.956	27.92	73.43
WM	FFT	1.4 M	5.444	42.63	119.25
	ATT	1.4 M	5.624	39.63	105.21
FZ	FFT	3.3 M	13.212	23.69	64.39
	ATT	3.5 M	14.192	37.38	97.14
KT	FFT	449 K	1.8	76.12	181.70
	ATT	454 K	1.820	70.20	174.08
MW	FFT	449 K	1.8	75.71	174.99
	ATT	454 K	1.820	71.10	168.50

**Figure A1:**
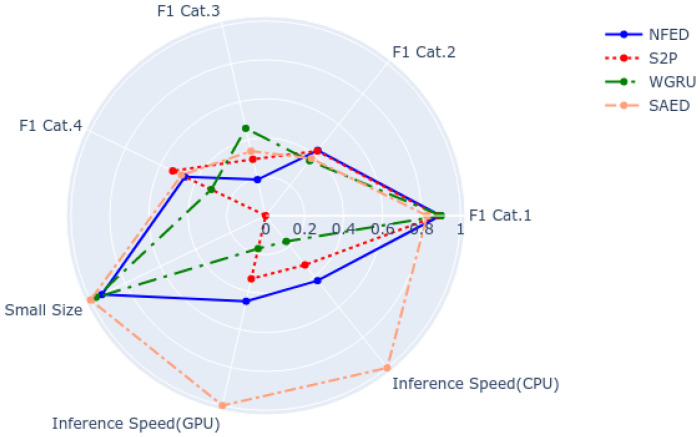
Diagram that summarizes the capabilities of the models to disaggregate a washing machine.

**Figure A2:**
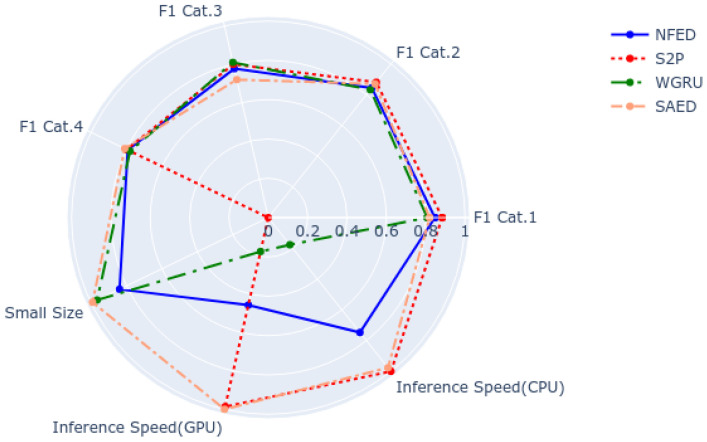
Diagram that summarizes the capabilities of the models to disaggregate a fridge.

**Figure A3:**
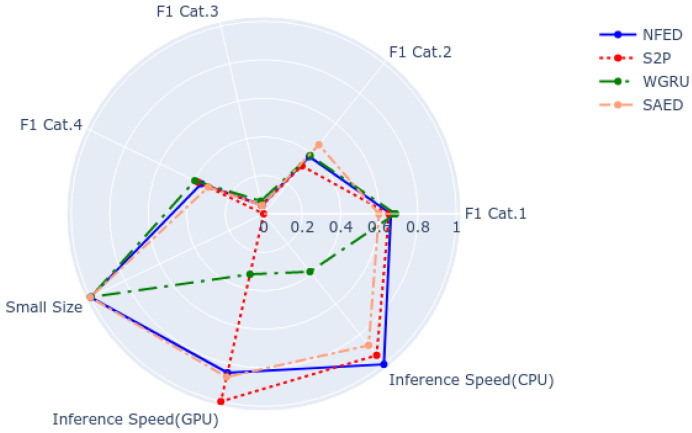
Diagram that summarizes the capabilities of the models to disaggregate a microwave.

**Figure A4:**
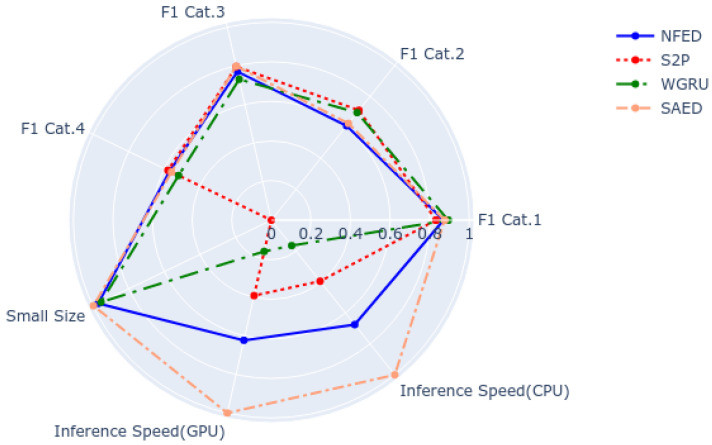
Diagram that summarizes the capabilities of the models to disaggregate a kettle.

## Data Availability

Not applicable.

## Appendix A

**Table A1:** Comparison of Classification and Estimation Accuracy between the models. Seen and Unseen houses are noted as *S* and *U* correspondingly. In the demonstrated case, AUH equals $F_{1}$ unseen and EUH equals MAE unseen.

Device	S|U	Model	$F_{1}$s	AUH	MGL [%]	MAEs	EUH [W]	MGL [%]
DW	1|2	NFED	0.69	0.34	51.7	5.7	21.62	279.2
		S2P	**0.71**	0.21	70.1	**5.12**	19.58	282.4
		WGRU	0.52	0.33	**37.5**	5.99	20.51	**242.4**
		SAED	0.44	0.26	41.0	7.69	29.88	288.7
WM	1|2	NFED	0.89	0.43	**52**	7.57	21.52	184.2
		S2P	0.88	0.42	**52**	8.05	20.71	157.3
		WGRU	**0.9**	0.36	59.9	**6.44**	17.22	167.4
		SAED	0.83	0.37	54.6	8.94	22.37	**150.3**
FZ	1|5	NFED	0.84	0.84	0.4	14.88	18.33	23.2
		S2P	**0.88**	0.88	0.3	**14.46**	16.64	15.1
		WGRU	0.81	0.83	−3.0	18.18	17.76	−2.3
		SAED	0.82	0.87	−6.1	18.59	16.62	−10.7
KT	1|2	NFED	0.87	0.61	29.9	4.25	12.87	202.8
		S2P	0.83	0.71	**14.7**	4.4	10.86	**146.7**
		WGRU	**0.89**	0.69	22.2	**4.1**	11.08	170.8
		SAED	0.87	0.62	28.6	4.81	12.93	168.8
MW	1|2	NFED	0.66	0.38	42.4	5.49	8.9	62.0
		S2P	0.65	0.32	51.1	5.01	5.67	13.2
		WGRU	**0.68**	0.39	43.3	**4.79**	5.98	24.7
		SAED	0.59	0.46	**23.2**	6.1	5.69	−6.5
